# Cellular Casein Kinase 2 and Protein Phosphatase 2A Modulate Replication Site Assembly of Bluetongue Virus*

**DOI:** 10.1074/jbc.M116.714766

**Published:** 2016-05-16

**Authors:** Bjorn-Patrick Mohl, Polly Roy

**Affiliations:** From the Department of Pathogen Molecular Biology, Faculty of Infectious and Tropical Diseases, London School of Hygiene and Tropical Medicine, Keppel Street, London WC1E 7HT, United Kingdom

**Keywords:** phosphorylation, protein phosphatase 2 (PP2A), viral protein, viral replication, virus assembly, bluetongue virus, CK2, VIBs, virus inclusion bodies

## Abstract

A number of cytoplasmic replicating viruses produce cytoplasmic inclusion bodies or protein aggregates; however, a hallmark of viruses of the Reoviridae family is that they utilize these sites for purposes of replication and capsid assembly, functioning as viral assembly factories. Here we have used bluetongue virus (BTV) as a model system for this broad family of important viruses to understand the mechanisms regulating inclusion body assembly. Newly synthesized viral proteins interact with sequestered viral RNA molecules prior to capsid assembly and double-stranded RNA synthesis within viral inclusion bodies (VIBs). VIBs are predominantly comprised of a BTV-encoded non-structural protein 2 (NS2). Previous *in vitro* studies indicated that casein kinase 2 (CK2) mediated the phosphorylation of NS2, which regulated the propensity of NS2 to form larger aggregates. Using targeted pharmacological reagents, specific mutation in the viral genome by reverse genetics and confocal microscopy, here we demonstrate that CK2 activity is important for BTV replication. Furthermore, we show that a novel host cell factor, protein phosphatase 2A, is involved in NS2 dephosphorylation and that, together with CK2, it regulates VIB morphology and virus replication. Thus, these two host enzymes influence the dynamic nature of VIB assembly/disassembly, and these concerted activities may be relevant to the assembly and the release of these cores from VIBs.

## Introduction

Reversible protein phosphorylation is the most pervasive mechanism within cells to facilitate the continual adjustment of anabolic, catabolic, and signal transduction events to maintain cellular equilibrium (1). In eukaryotes, protein kinases catalyze the transfer of the γ-phosphate from ATP predominantly to serine, threonine, and tyrosine residues (2, 3). Conversely, protein phosphatases catalyze the removal of such negatively charged phosphoryl moieties, orchestrating dephosphorylation in highly regulated kinase-phosphatase dynamics and networks (4, 5). The addition or removal of such negatively charged phosphoryl moieties can influence protein function through conformational changes that alter the affinity for ligands, stability, or subcellular localization (6). Certain RNA viruses, such as HIV, type 1 (7), Ebola virus (8), vesicular stomatitis virus (9), rubella virus (10), and hepatitis C virus (11), utilize protein phosphorylation as a crucial mechanism for the regulation of viral RNA binding activity, transcription, replication, and virus assembly. For the members of Reoviridae, it is the virally encoded non-structural proteins that are phosphorylated, such as rotavirus NSP5 (12) and involved in many of these activities during the virus life cycle. However, these do not constitute components of the mature virus particles.

Bluetongue virus (BTV),^2^ the prototype member of the *Orbivirus* genus in the Reoviridae family, is an insect-vectored emerging pathogen of wild ruminants and livestock (with mortality reaching 70% in sheep) in many parts of the world. BTV is an icosahedral double-capsid virus, and the virion particle is an architecturally complex structure. It is composed of seven structural proteins (VP1–VP7) organized in two concentric protein shells surrounding a genome of 10 segmented double-stranded RNAs. In addition to seven structural proteins, four non-structural proteins (NS1–NS4) are also synthesized in the infected host cells. Of the 11 viral proteins synthesized during BTV infection, only the nonstructural protein 2 (NS2) is phosphorylated (13, 14) and is also the principal component of cytoplasmic viral inclusion bodies (VIBs), the site of viral assembly (15). NS2 is phosphorylated at Ser-249 and Ser-259 (16). NS2 expressed singly or in the context of an infection amalgamates in the cytoplasm to form globular aggregates and acts as a scaffold or concentrator in the cytoplasm for viral RNAs and proteins.

NS2 is responsible for recruiting the newly synthesized core (inner capsid) components, including the core proteins and 10 single-stranded RNA transcripts into the VIBs, where core assembly occurs (16, 17). Furthermore, although NS2 is not a component of the mature virus, it is indispensable for the assembly of the primary replicase complex to initiate secondary replication in the infected host cells (18).

Because our previous studies suggested that cellular CK2 is responsible for NS2 phosphorylation (16), we have undertaken further examinations of whether CK2 activity is indeed important for BTV replication and whether a phosphatase activity complements the activity of the kinase (CK2), mediating NS2 phosphorylation dynamics. Accumulating data obtained from a series of *in vivo* studies using various specific pharmacological inhibitors and enhancers demonstrated that CK2 activity is important for BTV replication. Furthermore, we identified a novel NS2 interaction partner, protein serine/threonine phosphatase type 2A (PP2A), whose activity also appeared to be important for BTV replication.

## Results

### 

#### 

##### CK2 Activity Is Important for Viral Replication and for Regulating VIB Morphology during BTV Infection

**FIGURE 1.**
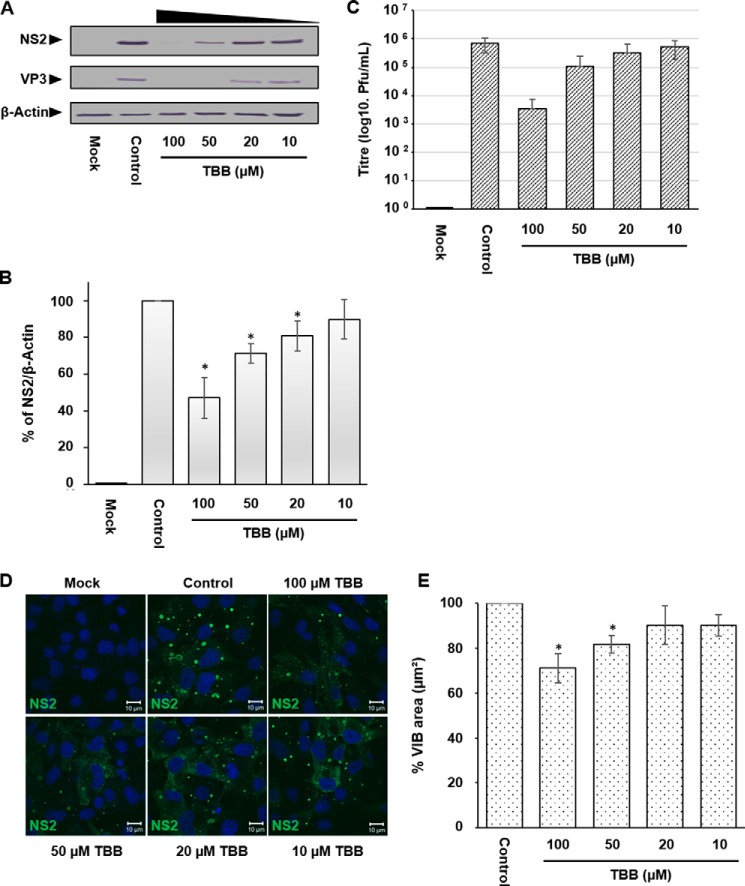
**CK2 activity is important for viral replication and for regulating VIB morphology during BTV infection.** HeLa cells infected with BTV1 (MOI = 1) were treated 4 h.p.i. with concentrations of 100, 50, 20, and 10 μm TBB for 20 h. *A*, samples were analyzed by Western blotting using specific antibodies as indicated. *B*, densitometry analysis of the Western blots expressed as a percentage. *C*, virus titer derived from HeLa cells infected with BTV1 (MOI = 1) and treated 4 h.p.i. with concentrations of 100, 50, 20, and 10 μm TBB for 20 h. *D*, HeLa cells infected with BTV1 (MOI = 1) were treated 20 h.p.i. with concentrations of 100, 50, 20, and 10 μm TBB for 4 h. *E*, VIBs denoted by NS2 staining were quantified using Volocity software, and their size was determined in replicate experiments of *D. Error bars* represent the standard deviation (Western blots) and standard error (confocal images) values of stimulations from three independent experiments. *, *p* < 0.05.

The phosphorylation state of two serine residues at positions 249 and 259 within NS2 regulates its capacity to amalgamate and form large VIBs (16); however, non-phosphorylated NS2 retains the capacity to oligomerize and form small aggregates (16, 19, 20). Previous studies implicated that the cellular kinase CK2 mediates this modification independent of infection (16). To obtain direct evidence that CK2 activity is important for virus replication by regulating NS2 phosphorylation, we used a specific inhibitor for CK2, 4,5,6,7-tetrabromobenzotriazole (TBB) (21), an ATP/GTP-competitive inhibitor of CK2. HeLa cells were infected with BTV1 (MOI = 1) for 4 h prior to treatment with TBB at a gradient of concentrations (100–10 μm) for 20 h. Subsequent Western blotting analysis showed that TBB treatment interfered with viral replication, decreasing both NS2 and VP3 protein levels (Fig. 1*A*). When NS2 protein levels were normalized to host cell β-Actin levels via densitometry in replicate experiments, TBB (100, 50, and 20 μm) significantly decreased BTV1 replication compared with the control (Fig. 1*B*). 100 μm TBB decreased NS2 protein levels by ∼60% (± 11%), 50 μm by ∼30% (± 5%), and 20 μm by ∼20% (± 8%). Moreover, we also observed an ∼2 log10 and ∼1 log10 decrease in virus titer in virus derived from cells treated with 100 and 50 μm (Fig. 1*C*).

To investigate the effect on VIB morphology, cells were treated with a gradient of TBB concentrations (100–10 μm) for 4 h and analyzed by immunofluorescence confocal microscopy. As a result of CK2 inhibition, VIB morphology was altered and appeared smaller than the normal VIBs. Furthermore, NS2 was also more dispersed throughout the cytoplasm when treated with TBB (Fig. 1*D*). To quantify VIB size, we calculated the surface area of VIBs. Immunofluorescence images were collated using Volocity software. The quantification data revealed that treatment of virus-infected cells with 100 and 50 μm TBB significantly decreased mean VIB size (square micrometers) by ∼30% (± 6%) and ∼20% (± 4%), respectively (Fig. 1*E*).

**FIGURE 2.**
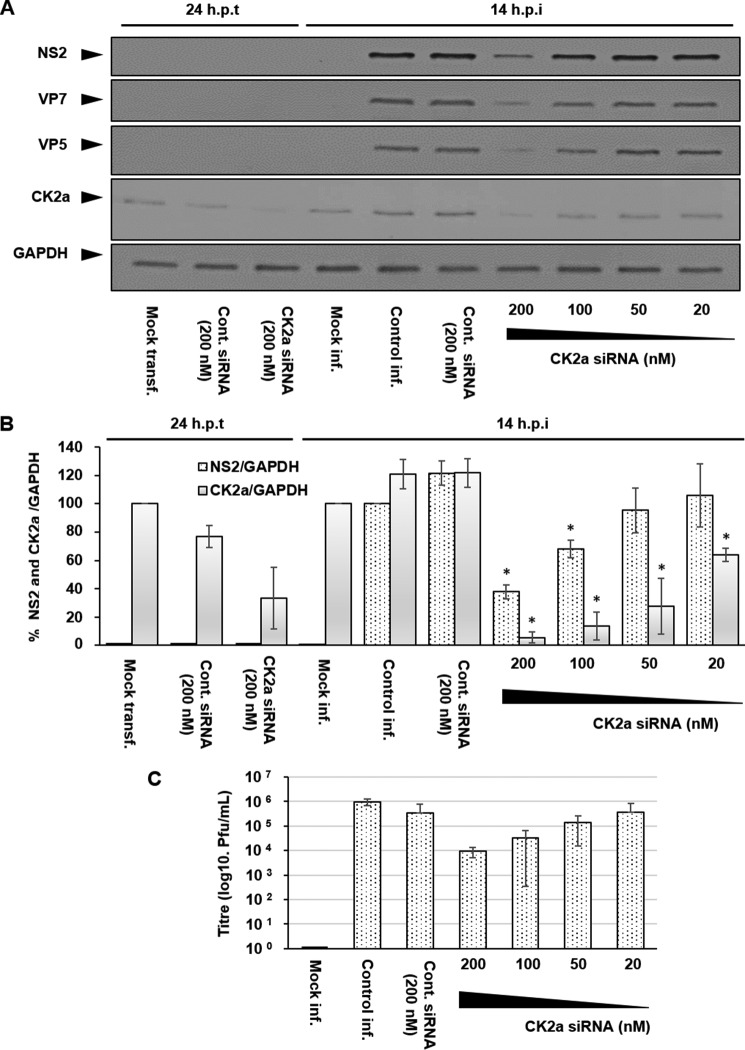
**siRNA knockdown of CK2a inhibits BTV replication.** HeLa cells were either mock-transfected (*mock transf.*) or transfected with 200 nm control (*cont.*) siRNA or a gradient of CK2a siRNA at concentrations (200–20 nm) for 24 h. Transfected cells were then infected (*inf.*) with BTV1 (MOI = 1) for 14 h. *A*, samples were analyzed by Western blotting using specific antibodies as indicated. *B*, densitometry analysis of the Western blots are shown expressed as a percentage representing NS2 (*dotted columns*) and CK2a (*solid columns*) protein levels relative to GAPDH. *C*, virus titer derived from HeLa cells 14 h.p.i. *Error bars* represent the standard deviation values of stimulations from three independent experiments. *, *p* < 0.05.

**FIGURE 3.**
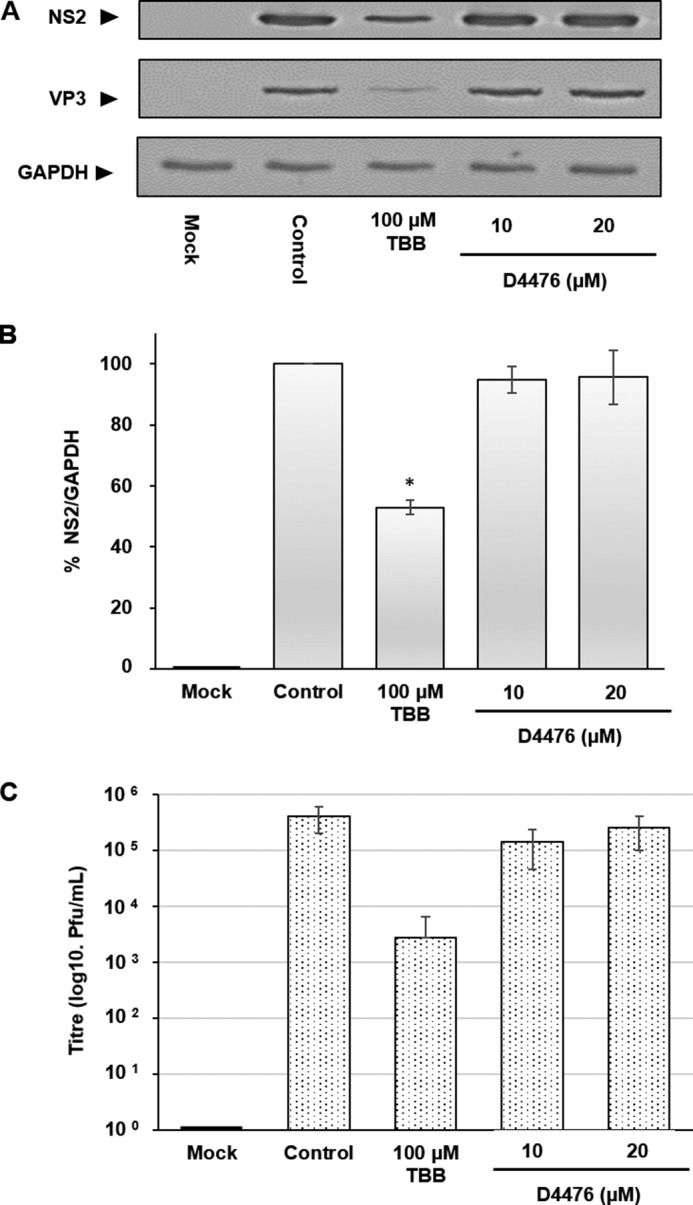
**Inhibition of CK1 does not inhibit BTV replication.** HeLa cells infected with BTV1 (MOI = 1) were treated 4 h.p.i. with 100 μm TBB and 10 and 20 μm of D4476 for 20 h. *A*, samples were analyzed by Western blotting using specific antibodies as indicated. *B*, densitometry analysis of the Western blots expressed as a percentage. *C*, virus titer derived from HeLa cells infected with BTV1 (MOI = 1) and treated 4 h.p.i. with the indicated compound and concentration. *Error bars* represent the standard deviation values of stimulations from three independent experiments. *, *p* < 0.05.

To support the data obtained from CK2 inhibition, we subsequently undertook siRNA knockdown experiments targeting CK2a that contains the catalytic subunit of the kinase tetramer. HeLa cells were mock-transfected or transfected with either a control siRNA (200 nm) or CK2a siRNA at different concentrations (200–20 nm) for 24 h prior to infection. Samples at 24 h post-transfection were taken to confirm the successful knockdown of CK2a protein levels prior to infection. Cells were then infected with BTV1 at MOI 1 and harvested 14 h post-infection. Lysates were then analyzed by Western blotting using specific antibodies for each protein (Fig. 2*A*). Quantification of Western blotting via densitometry confirmed that, at the time of infection (24 h post-transfection), CK2a protein levels had decreased by ∼65% (± 20%). Subsequent to infection, we observed a concentration-dependent decrease in NS2 protein levels that correlated to the corresponding decreases in CK2a protein levels. The level CK2a protein at 14 h.p.i. was decreased by ∼90% (± 5%) in the presence of 200 nm siRNA. This decrease correlated with a ∼62% decrease (± 6%) in NS2 protein levels. Similarly, 100 nm siRNA also decreased CK2a protein levels ∼85% (± 10%). This decrease correlated with a ∼32% decrease (± 6%) in NS2 protein levels (Fig. 2*B*). Furthermore, 200 and 100 nm siRNA decreased the virus titer by ∼2 log10 and ∼1 log10, respectively (Fig. 2*C*). To examine whether CK1 activity was as important for BTV replication as CK2, we tested the effect of a specific CK1 inhibitor, D4476 (22). CK1 inhibition did not result in any significant decreases in viral protein levels at the indicated concentrations (Fig. 3, *A* and *B*), nor did it have any effect on virus titers (Fig. 3*C*) compared with the CK2 inhibitor TBB, which served as a positive inhibition control. Cumulatively, these data confirm that CK2 but not CK1 activity is important for BTV replication.

##### Identification of Protein Serine/Threonine PP2A as a novel NS2 Interaction Partner Associated with the Dephosphorylation of NS2

**FIGURE 4.**
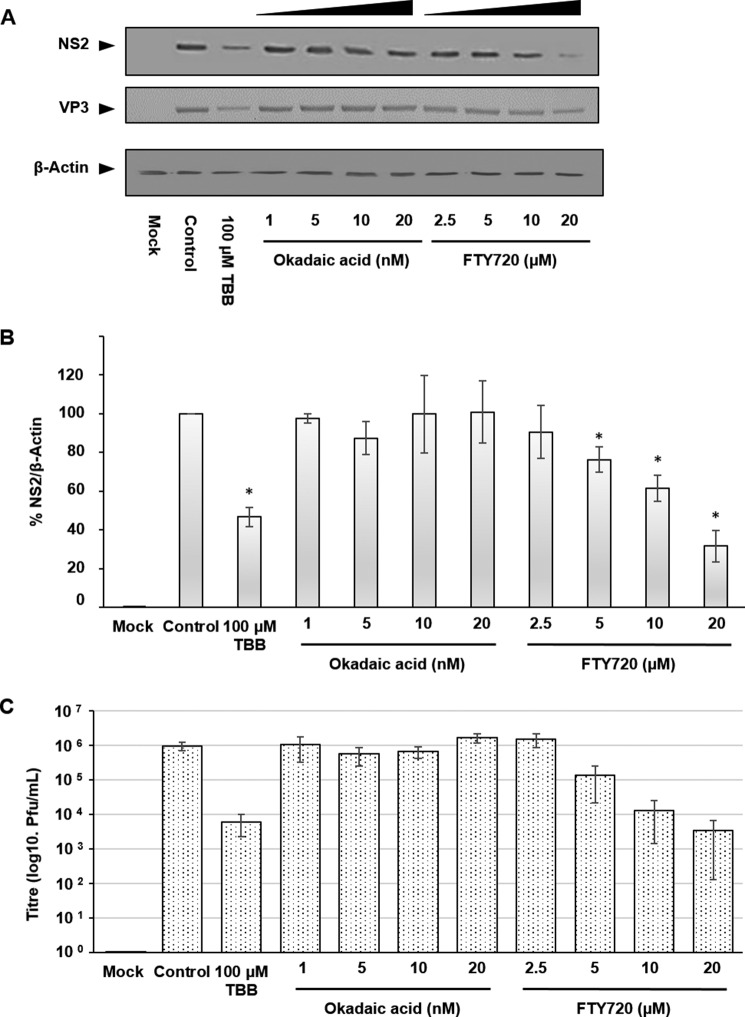
**PP2A activity affects viral replication.** HeLa cells infected with BTV1 (MOI = 1) were treated 4 h.p.i. with TBB (a CK2 inhibitor), okadaic acid (an inhibitor of PP2A), and FTY720 (an activator of PP2A) for 20 h at the indicated concentrations. *A*, samples were analyzed by Western blotting using specific antibodies as indicated. *B*, densitometry analysis of the Western blots expressed as a percentage. *C*, virus titer derived from HeLa cells infected with BTV1 (MOI = 1) and treated 4 h.p.i. with the indicated compound and concentration. *Error bars* represent the standard deviation values of stimulations from three independent experiments. *, *p* < 0.05.

It is likely that the phosphorylation state of NS2 is a dynamic process and that it is co-regulated by a complementary phosphatase that could function in concert with CK2, thereby controlling NS2 phosphorylation dynamics. CK2 was previously found to directly interact with PP2A and act in concert (23), making it a plausible candidate for our study. To explore the potential role of PP2A, we tested a PP2A inhibitor, okadaic acid (24), and an activator, FTY720 (25). HeLa cells were infected with BTV1 at MOI 1 and, at 4 h.p.i., treated either with a gradient of concentrations of okadaic acid (1–20 nm) or FTY720 (2.5–20 μm) for 20 h. As a replication inhibition control, 100 μm TBB treatment was also included. Subsequent Western blotting analysis showed that 5, 10, and 20 μm FTY720 interfered with viral replication, decreasing both NS2 and VP3 protein levels, similar to the effect of the control TBB treatment that had been observed previously. Okadaic acid treatment did not result in reduction of NS2 and VP3 protein levels (Fig. 4*A*). When NS2 protein levels were normalized to β-Actin levels via densitometry in replicate experiments, FTY720 significantly decreased replication compared with the untreated control (Fig. 4*B*). At 20 μm, FTY720 decreased NS2 protein levels by almost ∼70% (± 8%) and at 10 and 5 μm by ∼40% (± 7%) and ∼25% (± 7%), respectively, whereas 100 μm TBB decreased NS2 protein levels by over 50%.

To further support our observations, we examined the effects of different concentrations of these drugs on virus growth. Cells were infected and treated as above. Lysates were then used for plaque assays. Consistent with the Western blotting data, virus titers in FTY720-treated cells also decreased, ∼2.5 log10 at 20 μm, ∼2 log10 at 10 μm, and ∼1 log10 at 5 μm, respectively, correlating with the decreased viral protein levels (Fig. 4*C*).

##### CK2 Inhibition or PP2A Activation Decreases NS2 Phosphorylation

**FIGURE 5.**
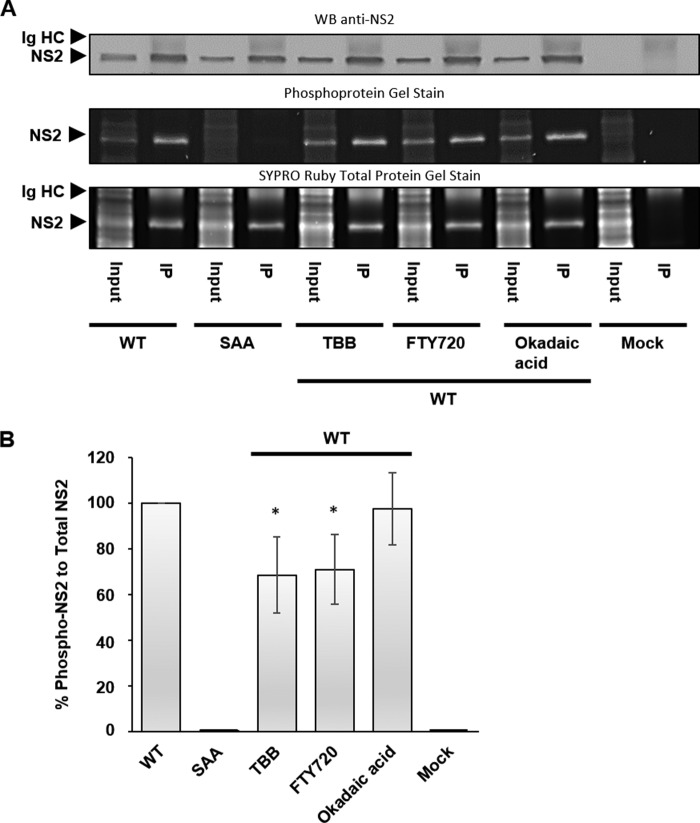
**NS2 phosphorylation at Ser-249 and Ser-259 decreases following CK2 inhibition or PP2A activation.** HeLa cells infected with BTV1 (MOI = 1) were treated 20 h.p.i. for 4 h with 100 μm TBB, 10 μm FTY720, and 20 nm okadaic acid. NS2 was isolated from these cells using immunoprecipitation (*IP*). *A*, samples were analyzed by Western blotting to confirm NS2 identity. SDS-PAGE gels were sequentially stained for phosphoproteins and total proteins. *B*, densitometry analysis of phosphoprotein and total protein staining data in *A* from replicate experiments. NS2 is indicated along the detected Ig heavy chains (*HC*). *Error bars* represent the standard deviation values of stimulations from three independent experiments. *, *p* < 0.05.

To obtain direct evidence that CK2 or PP2A affect NS2 phosphorylation, we quantified the phosphorylation state of NS2 following CK2 and PP2A activity modulation. HeLa cells were infected with BTV1 for 20 h before treatment with either 100 μm TBB, 10 μm FTY720, or 20 nm okadaic acid for 4 h. Cells were lysed, and NS2 was purified by immunoprecipitation. The presence of NS2 in the immunoprecipitation fractions was verified using Western blotting (Fig. 5*A*). A duplicate SDS-PAGE gel was stained sequentially with Pro-Q Diamond phosphoprotein gel stain and SYPRO Ruby protein gel stain to determine protein phosphorylation and total protein concentrations, respectively (Fig. 5*A*). Fluorescence quantification of the acquired images allowed for the generation of phosphoprotein to total protein ratios normalized to WT virus (Fig. 5*B*). For a negative control, we generated a NS2 phosphorylation mutant virus, where the previously identified phosphorylated serine sites (Ser-249 and Ser-259) were substituted by alanine residues (SAA) using a reverse genetics system of BTV (16, 26).

The phosphoprotein-stained gel showed WT NS2 phosphorylated, but the SAA mutant lacking these phosphorylation sites was not affected. This supports previous observations that Ser-249 and Ser-259 are the principal sites of phosphorylation within NS2. NS2 in the immunoprecipitation fractions from WT, SAA, 100 μm TBB-treated, 10 μm FTY720-treated, and 20 nm okadaic acid-treated cells was quantified. Phosphoprotein to total protein ratios showed that, when CK2 activity was inhibited following TBB treatment, NS2 phosphorylation significantly decreased by ∼30% (± 16%). Similarly, when PP2A was activated using FTY720, NS2 phosphorylation significantly decreased by ∼30% (± 15%). However, when PP2A activity was inhibited following okadaic acid treatment, no statistically significant change could be observed (Fig. 5*B*).

##### CK2 and PP2A Co-regulate VIB Morphology

**FIGURE 6.**
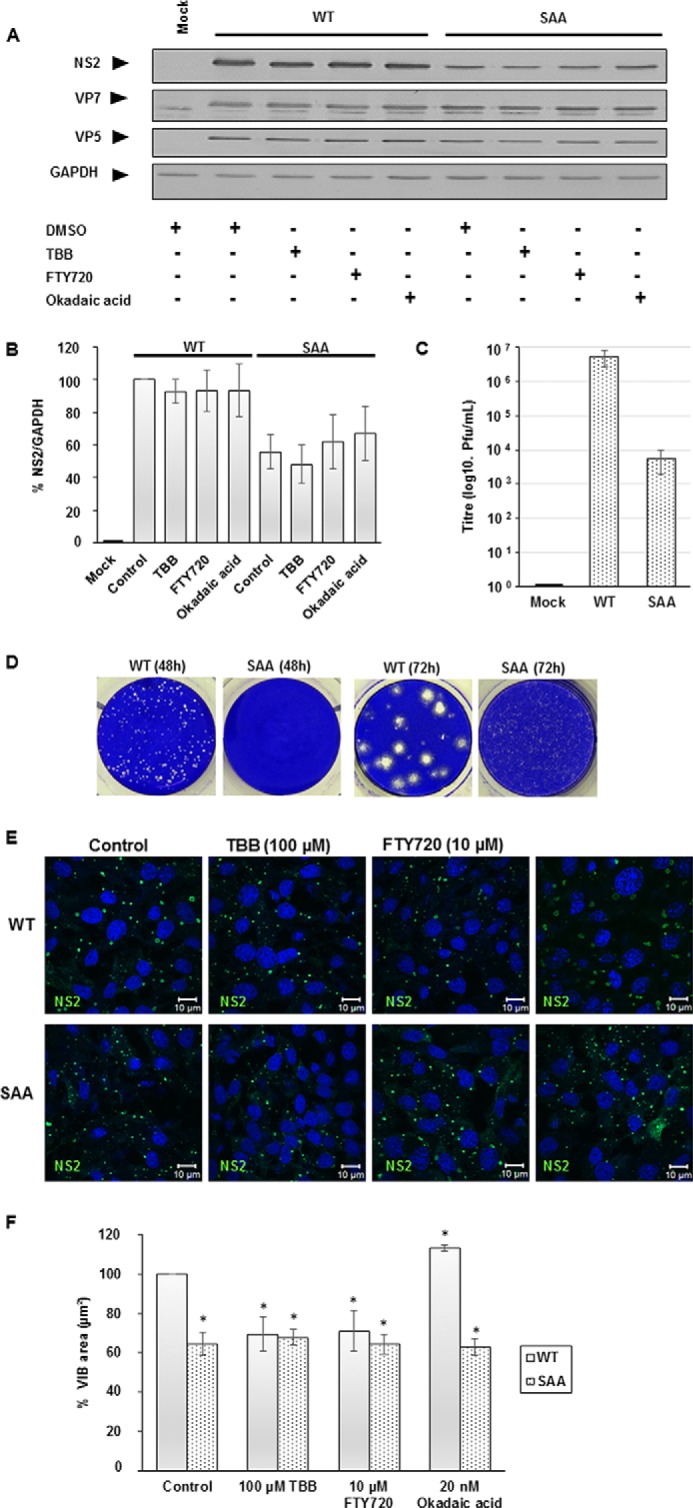
**Transient treatment with CK2 inhibitor, PP2A inhibitor, or activator modify VIB morphology in the WT virus but not the alanine mutant virus.** HeLa cells infected with BTV1 (MOI = 1) were treated 20 h.p.i. for 4 h with 100 μm TBB, 10 μm FTY720, or 20 nm okadaic acid. *A*, samples were analyzed by Western blotting using specific antibodies as indicated. *B*, densitometry analysis of the Western blots expressed as a percentage. *C*, virus titer derived from untreated HeLa cells infected with BTV1 WT and SAA mutant virus (MOI = 1). *D*, plaque assay plates of WT and SAA mutant virus at 48 and 72 h.p.i. *E*, HeLa cells infected with BTV1 WT and SAA mutant virus (MOI = 1) were treated 20 h.p.i. with 100 μm TBB, 10 μm FTY720, or 20 nm okadaic acid for 4 h. *F*, VIBs denoted by NS2 staining of WT (*solid columns*) and SAA mutant (*dotted columns*) virus were quantified using Volocity software, and their size was determined in replicate experiments of *E. Error bars* represent the standard deviation (Western blots) and standard error (confocal images) values of stimulations from three independent experiments. *, *p* < 0.05.

To further investigate the effect of CK2 inhibition or PP2A activation on VIB morphology, BTV-infected (WT or SAA) cells were transiently treated with each pharmacological reagent. HeLa cells were infected for 20 h before treatment with either 100 μm TBB, 10 μm FTY720, or 20 nm okadaic acid for a further 4 h to minimize any potential NS2 protein turnover. Samples were probed for NS2, VP7, VP5, and GAPDH (Fig. 6*A*).

Densitometry analysis of Western blots of each sample, both for the WT and the SAA mutant viruses, showed no significant decrease of NS2 protein levels following this transient drug treatment (Fig. 6, *A* and *B*). However, the SAA mutant virus displayed a replication deficiency, with NS2 protein levels appreciably lower than those of the WT. Plaque assays showed that, compared with the WT, the SAA mutant virus displayed replication impairment with a titer of ∼3 log10 lower than that of the WT (Fig. 6*C*). Furthermore, the plaque morphology profile differed, with the SAA mutant virus forming smaller plaques compared with the WT at corresponding time points post-infection (Fig. 6*D*).

Complementary immunofluorescence showed that VIBs of WT virus were smaller and NS2 was more dispersed throughout the cytoplasm when infected cells were treated with TBB and FTY720 but larger when treated with Okadaic acid, following this transient treatment. Concurrently, the SAA mutant virus produced smaller VIBs that appeared unresponsive to the treatment (Fig. 6*E*). Quantification of immunofluorescence images revealed that TBB treatment of WT virus decreased the mean size (square micrometers) of VIB by ∼30% (± 9%). Similarly, activation of PP2A via FTY720 resulted in a decrease of mean VIB size by ∼30% (± 10%). When PP2A was inhibited using okadaic acid, the mean size of VIBs increased by ∼13% (± 2%). Although WT VIBs demonstrated these morphological changes, VIBs of the SAA mutant virus VIBs showed no significant change in size under the same conditions. Furthermore, the VIBs of the SAA mutant virus were significantly smaller (∼40% ± 6%) compared with VIBs of WT virus (Fig. 6*F*). Cumulatively, these data provide evidence for PP2A activity influencing NS2 phosphorylation and, similar to CK2, affecting VIB morphology (formation and size) and virus replication independent of NS2 protein levels.

## Discussion

BTV-induced VIBs, the site of viral replication and virus assembly, are primarily composed of NS2 and are essential for primary replication *in vivo* (17, 18). Although our previous study had indicated the involvement of CK2 in the modification of NS2 phosphorylation (16), the importance of CK2 activity in BTV replication remained to be validated. Furthermore, a correlating phosphatase and the dynamics that NS2 phosphorylation might orchestrate and control VIB assembly *in vivo* remained unknown. Our studies here confirmed that CK2 activity is in fact important for BTV replication *in vivo* and that PP2A co-regulates NS2 phosphorylation. However, in contrast, CK1 inhibition did not impair BTV replication or influence virus titer (Fig. 3, *A–C*) as opposed to CK2 inhibition. We have confirmed that the inhibition of CK2 activity via a pharmacological inhibitor, TBB, during transient treatment affected NS2 dephosphorylation (Fig. 5) and mediated a decrease in VIB size (Fig. 1, *D* and *E*). The size changes may be due to the VIB NS2 matrix disassembly because changes in the phosphorylation state influence the capacity of NS2 to aggregate (16) and thereby cause NS2:NS2 dissociation. Furthermore, prolonged treatment resulted in inhibition of virus replication and decreased virus titer in infected cells (Fig. 1, *A–C*). Similarly, siRNA knockdown of CK2a inhibited BTV replication and decreased virus titer (Fig. 2, *A–C*). These data also validate previous *in vitro* observations, in this case highlighting the importance of NS2 phosphorylation within the context of a productive viral replication cycle both for VIB formation as well as production of infectious virus particles. Consistent with our previous report (16), our data here show that CK2 influences NS2 phosphorylation *in vivo* and supports a direct relationship between NS2 phosphorylation, VIB formation, and size as well as virus replication.

Expanding on the known phosphorylation dynamics of NS2, we have also identified, for the first time, a phosphatase that appeared to co-regulate phosphorylation of NS2 alongside CK2. The PP2A holoenzyme is a heterotrimeric complex of a scaffolding A subunit, a regulatory B-type subunit, and a catalytic C subunit (27, 28). Although there are currently no known consensus sequences in PP2A substrates (29), pharmacological interventions specifically targeting PP2A resulted in NS2 dephosphorylation and triggered NS2 phosphorylation-specific morphological changes in VIBs. Similar observations were made in a sheep cell line, a natural host species (data not shown).

Thus, several lines of evidence corroborate that PP2A constitutes a component of the regulatory mechanism that modulates NS2 phosphorylation dynamics alongside CK2. First, NS2 phosphorylation levels decreased during the activation of PP2A following FTY720 treatment to similar levels observed when CK2 was inhibited using TBB. Second, VIB morphology in cells proved responsive to a pharmacological inhibitor and activator that targets PP2A activity. Pharmacological inhibition of PP2A using okadaic acid resulted in an increase in VIB size, whereas, conversely, pharmacological activation of PP2A using FTY720 reduced VIB size.

Furthermore, by utilizing a mutant virus lacking phosphorylation sites (alanine substitutions at Ser-249 and Ser-259), we showed that this virus displayed an impaired replication profile compared with WT virus. Although we also documented that the phosphorylation state of Ser-249 and Ser-259 could be modulated and that VIB morphology was responsive to CK2 inhibition and PP2A inhibition and activation in WT virus, the SAA mutant remained unresponsive to such modulation. This was clearly shown when pharmacological treatments did not elicit any significant changes in VIB size.

However, it cannot be ruled out that other kinases or phosphatases are involved in the regulation of NS2 phosphorylation and dephosphorylation. This was evidenced during prolonged CK2 inhibition using TBB and siRNA knockdown of CK2a or, similarly, prolonged activation of PP2A using FTY720. None of these interventions could completely abolish BTV replication or the production of infectious virus, although the decrease was significant in each case. However, TBB, FTY720 and okadaic acid were added post-entry (4 h.p.i.); thus, they could not impair initial replication. Similarly, during the siRNA knockdown experiment, some CK2a was still present. In each case, this may have been sufficient for initial replication and infection to be established. Overall, it should be noted that the SAA mutant virus that lacked the NS2 phosphorylation sites while virus growth was significantly impaired still retained a low-level capacity for replication, much like the WT virus treated with CK2 inhibitor or PP2A activator.

Furthermore, the reduction of NS2 phosphorylation during transient treatment of cells with the inhibitors and activator achieved an ∼30% decrease in phosphorylation. This could indicate the existence of other NS2 phosphorylation regulators. A further possible reason for this observation could be the transient nature of the treatment. Similarly, this could be a result of enzyme kinetics or varying inhibitor efficiencies.

Within the context of the virus life cycle, this dynamic nature of NS2 phosphorylation may facilitate more than VIB assembly. Although the inner capsids (cores) of all members of the Reoviridae are assembled within inclusion bodies, the assembly of the outer capsid and virion maturation take place outside of these structures. Thus, the timely release of cores from VIBs is an essential step in virion morphogenesis that must be regulated. As cores traffick out of the virus inclusion bodies to mature, the NS2 phosphorylation we have described could plausibly serve as the key regulator. Functionally, phosphorylation of NS2 would modify the morphology of VIBs to allow maturation to take place, with the balance of CK2 and PP2A activities acting as the regulatory mechanism that facilitates this, representing another case of host-virus interaction that relates to virus infection and pathogenesis.

## Experimental Procedures

### 

#### 

##### Cell Lines and Virus

BSR cells (BHK-21 subclone, BHK21 cells (ATCC, CCL10^TM^) and HeLa cells (HeLa, ATCC, CCL-2^TM^)) were maintained in DMEM (Sigma-Aldrich) supplemented with 10% (v/v) FBS (Invitrogen), 100 units of penicillin/ml, and 100 μg of streptomycin/ml (Sigma-Aldrich) and minimum Eagle's medium non-essential amino acids (Gibco).

BTV serotype 1 (BTV1) stock was obtained by infecting BSR cells at a low multiplicity of infection and harvested when a 100% cytopathic effect was evident. Virus stocks were stored at 4 °C. The BTV1 double alanine mutant (SAA) was generated using the previously described reverse genetics system (18).

##### Pharmacological Reagents

The CK2 inhibitor TBB, the PP2A activator FTY720, and the PP2A inhibitor okadaic acid used were purchased from Santa Cruz Biotechnology, and the CK1 inhibitor D4476 was purchased from Sigma-Aldrich. All reagents were used at the concentrations specified.

##### Immunofluorescence Microscopy

HeLa cells were grown on glass coverslips to 90% confluence prior to infection with BTV1 (MOI = 1). At specified times, cells were washed with PBS before being fixed for 10 min in 4% paraformaldehyde. Cells were permeabilized with ice-cold methanol for 10 min. Coverslips were washed with PBS and blocked with 1% BSA in PBS for 1 h at room temperature. Coverslips were incubated with the primary anti-NS2 antibody (guinea pig anti-NS2 serum) for 1 h at room temperature and washed in PBS before being incubated with secondary antibody (goat anti-guinea pig IgG (H+L) secondary antibody, Alexa Fluor 488 conjugate, A-11073, Thermo Fisher) and Hoechst 33342 (Invitrogen) for 1 h at room temperature. Coverslips were washed with PBS before being mounted on slides on mounting medium (Invitrogen).

##### Volocity High-performance Three-dimensional Imaging Software

Lsm files were uploaded into Volocity software (PerkinElmer Life Sciences). Data were analyzed using software algorithms.

##### siRNA Knockdown

HeLa cells were transfected with 200 nm Silencer® negative control 1 siRNA (AM4611, Ambion) or with SignalSilence® CK2α siRNA I (6389, Cell Signaling Technologies) at different concentrations (200–20 nm) using Lipofectamine® RNAiMAX transfection reagent (13778-100, Invitrogen). Transfections were carried out according to the instructions of the supplier. Cells were transfected for 24 h prior to BTV1 (MOI = 1) infection. Samples were harvested at the indicated time points for Western blotting analysis.

##### Western Blotting Analysis

SDS-PAGE gels were transferred via a semidry blotter to PVDF transfer membranes and blocked for 4 h with Tris-buffered saline containing 0.05% Tween 20 and 10% (w/v) milk powder. The following primary antibodies were used for the detection of: NS2 (guinea pig anti-NS2 serum), VP3 (rabbit anti-VP3 serum), β-Actin (monoclonal anti-β-Actin antibody produced in mouse, clone AC-74, A2228, Sigma-Aldrich), GAPDH (rabbit anti-GAPDH (ab9485, Abcam), anti-CKII α antibody (ab10466, Abcam). These were added to blocked membranes and incubated overnight at 4 °C. Secondary antibodies were an alkaline phosphatase-conjugated goat anti-guinea pig IgG (1:10,000, Sigma-Aldrich, A5062), goat anti-rabbit (A0418), and an alkaline phosphatase-conjugated goat anti-mouse (A3562) IgG (1:10,000, Sigma-Aldrich).

##### Immunoprecipitation

NS2 was purified from BTV1-infected HeLa cells (MOI = 1) after 24 h. Cells were washed with PBS before lysis. Cells were lysed in lysis buffer (50 mm Tris-HCl (pH 7.5), 125 mm NaCl, 5% Glycerol, 0.2% Nonidet P-40, 1.5 mm MgCl_2_, 25 mm NaF, 1 mm Na_3_VO_4_, and protease inhibitor) for 30 min on ice. Lysates were centrifuged at 5000 × *g* for 10 min. Supernatants were recovered and added to protein A-Sepharose beads (Rabbit anti-NS2, conjugated) and were incubated on ice overnight. Samples were centrifuged at 2000 × *g* for 10 min. The supernatant was removed, and the protein A-Sepharose beads washed with lysis buffer. Samples were centrifuged at 2000 × *g* for 10 min. This wash process was repeated four times. SDS loading buffer was then added to the protein A-Sepharose beads before being boiled.

##### Phosphoprotein Staining

SDS-PAGE gels were sequentially stained with Pro-Q Diamond phosphoprotein gel stain and SYPRO Ruby protein gel stain (Thermo Fisher). For each stain, the respective fluorescence was detected and quantified.

## Author Contributions

B. P. M. and P. R. designed the study and wrote the paper. B. P. M. designed, performed, and analyzed the experiments.

## Footnotes

2 The abbreviations used are:

BTV: bluetongue virus
VIB: viral inclusion body
PP2A: protein serine/threonine phosphatase type 2A
TBB: 4,5,6,7-tetrabromobenzotriazole
MOI: multiplicity of infection
h.p.i.: hours post-infection.
